# Di-μ-chlorido-bis­[(1,10-phenanthroline-κ^2^ *N*,*N*′)(trichloro­acetato-κ*O*)copper(II)]

**DOI:** 10.1107/S1600536812003947

**Published:** 2012-02-04

**Authors:** Gholam Hossein Shahverdizadeh, Seik Weng Ng, Edward R. T. Tiekink, Babak Mirtamizdoust

**Affiliations:** a Department of Chemistry, Faculty of Science, Tabriz Branch, Islamic Azad University, PO Box 1655, Tabriz, Iran; b Department of Chemistry, University of Malaya, 50603 Kuala Lumpur, Malaysia; c Chemistry Department, Faculty of Science, King Abdulaziz University, PO Box 80203, Jeddah, Saudi Arabia; d Department of Inorganic Chemistry, Faculty of Chemistry, University of Tabriz, PO Box 5166616471, Tabriz, Iran

## Abstract

The title compound, [Cu_2_(C_2_Cl_3_O_2_)_2_Cl_2_(C_12_H_8_N_2_)_2_], features a centrosymmetric binuclear complex. The coordination geometry around the Cu^II^ atom is square-pyramidal, comprising two N atoms from a symmetrically chelating 1,10-phenanthroline ligand, one O atom from a trichloro­acetate ligand and two Cl^−^ anions. In addition, there is a weak intra­molecular Cu⋯O inter­action of 2.9403 (14) Å involving the carbonyl O atom of the trichloro­acetate ligand. The central Cu_2_Cl_2_ core takes the form of a rhombus, owing to the disparate Cu—Cl bond lengths. Mol­ecules are connected in the crystal structure by C—H⋯Cl and C—H⋯O inter­actions.

## Related literature

For background to crystal engineering studies of Cu^II^ 1,10-phenanthroline complexes, see: De Burgomaster *et al.* (2010 ▶). For specialized crystallization techniques, see: Harrowfield *et al.* (1996 ▶). For closely related binuclear Cu^II^ mol­ecules with chloride, carboxyl­ate and bipyridine ligands, see: Jiang *et al.* (2007 ▶); Zheng *et al.* (2008 ▶). For descriptive parameters of pyramidal and trigonal–bipyramidal geometries, see: Addison *et al.* (1984 ▶); Spek (2009 ▶).
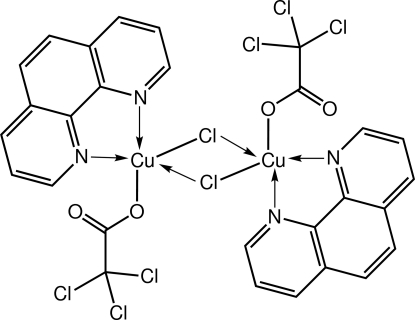


## Experimental

### 

#### Crystal data

[Cu_2_(C_2_Cl_3_O_2_)_2_Cl_2_(C_12_H_8_N_2_)_2_]
*M* *_r_* = 883.15
Monoclinic, 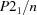

*a* = 9.2961 (2) Å
*b* = 17.3529 (2) Å
*c* = 10.6201 (2) Å
β = 115.269 (3)°
*V* = 1549.25 (5) Å^3^
*Z* = 2
Cu *K*α radiation
μ = 8.43 mm^−1^
*T* = 100 K
0.15 × 0.10 × 0.05 mm

#### Data collection

Agilent SuperNova Dual diffractometer with Atlas detector
Absorption correction: multi-scan (*CrysAlis PRO*; Agilent, 2010 ▶) *T*_min_ = 0.365, *T*_max_ = 0.678
11808 measured reflections
3233 independent reflections
3049 reflections with *I* > 2σ(*I*)
*R*_int_ = 0.019

#### Refinement

*R*[*F* ^2^ > 2σ(*F* ^2^)] = 0.026
*wR*(*F* ^2^) = 0.070
*S* = 1.05
3233 reflections
208 parameters
H-atom parameters constrained
Δρ_max_ = 0.52 e Å^−3^
Δρ_min_ = −0.46 e Å^−3^

### 

Data collection: *CrysAlis PRO* (Agilent, 2010 ▶); cell refinement: *CrysAlis PRO*; data reduction: *CrysAlis PRO*; program(s) used to solve structure: *SHELXS97* (Sheldrick, 2008 ▶); program(s) used to refine structure: *SHELXL97* (Sheldrick, 2008 ▶); molecular graphics: *ORTEP-3* (Farrugia, 1997 ▶) and *DIAMOND* (Brandenburg, 2006 ▶); software used to prepare material for publication: *publCIF* (Westrip, 2010 ▶).

## Supplementary Material

Crystal structure: contains datablock(s) global, I. DOI: 10.1107/S1600536812003947/bt5807sup1.cif

Structure factors: contains datablock(s) I. DOI: 10.1107/S1600536812003947/bt5807Isup2.hkl

Additional supplementary materials:  crystallographic information; 3D view; checkCIF report

## Figures and Tables

**Table 1:** Selected bond lengths (Å)

Cu—O1	1.9491 (13)
Cu—N1	2.0163 (16)
Cu—N2	2.0214 (16)
Cu—Cl1	2.2811 (5)
Cu—Cl1^i^	2.6666 (5)

Symmetry code: (i) 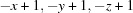.

**Table 2:** Hydrogen-bond geometry (Å, °)

*D*—H⋯*A*	*D*—H	H⋯*A*	*D*⋯*A*	*D*—H⋯*A*
C2—H2⋯Cl3^ii^	0.95	2.80	3.679 (2)	154
C4—H4⋯O2^iii^	0.95	2.49	3.302 (3)	144
C7—H7⋯Cl1^iv^	0.95	2.73	3.638 (2)	159

Symmetry codes: (ii) 
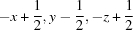
; (iii) 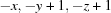; (iv) 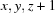.

**Fig. 1.**
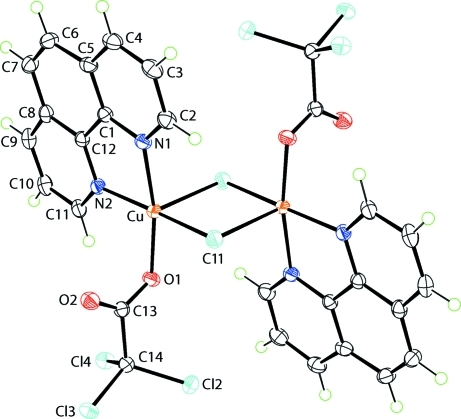
The molecular structure of (I) showing the atom-labelling scheme and displacement ellipsoids at the 50% probability level. Unlabelled atoms are related by the symmetry operation 1 - x, 1 - y, 1 - z.

**Fig. 2.**
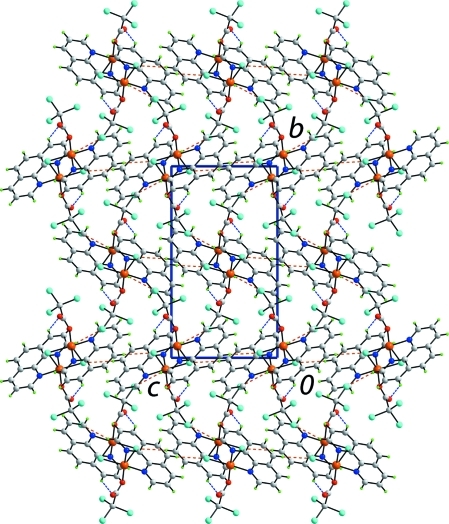
A view in projection down the a axis of the unit-cell contents for (I). The C—H···Cl and C—H···O interactions are shown as orange and blue dashed lines, respectively.

## supplementary crystallographic information

### Comment

Research on copper^II^ phenanthroline derivatives continues to attract interest in the context of crystal engineering of copper coordination polymers (De Burgomaster *et al.*, 2010). Herein, we report the title Cu^II^ complex, (I).

The Cu^II^ atom in binuclear (I), Fig. 1, is coordinated by two Cl atoms, which form dissimilar Cu—Cl bond lengths, two N atoms from a symmetrically chelating 1,10-phenanthroline ligand, and one O atom from a trichloroacetate ligand, Table 1. The structure of (I) is centrosymmetric and the central Cu_2_O_2_ has the form of a rhombus. The carbonyl-O2 atom forms a weak intramolecular Cu···O contact of 2.9403 (14) Å. The asymmetric mode of coordination of the carboxylate is reflected in the disparate C—O bond distances with the longer C13—O1 distance [1.270 (2) Å] being associated with the shorter Cu—O1 interaction, and the short C13—O2 distance [1.220 (2) Å] associated with the weaker Cu—O2 contact.

The resultant Cl_2_N_2_O donor set defines a square pyramid. This assignment is based on the value calculated for τ of 0.07 for the Cu atom, which compares to the τ values of 0.0 and 1.0 for ideal square pyramidal and trigonal bipyramidal geometries, respectively (Spek, 2009; Addison *et al.*, 1984). In this description, the less tightly bound Cl^i^ atom defines the axial site (*i*: 1 - *x*, 1 - *y*, 1 - *z*).

The observed coordination geometry in (I) resembles closely those found in the analogous structures with the carboxylate ligands being 2-anilinobenzoate (Jiang *et al.*, 2007) and *p*-tolylthioacetate (Zheng *et al.*, 2008).

In the crystal packing, molecules assembles into layers in the *ac* plane and are connected into the three dimensional architecture by C—H···Cl and C—H···O interactions, Fig. 2 and Table 2.

### Experimental

1,10-Phenanthroline (1 mmol) was placed in one arm of a branched tube (Harrowfield *et al.*, 1996) and a mixture of copper(II) chloride dihydrate (1 mmol) and trichloroacetic acid (1 mmol) in the other. Ethanol was then added to fill both arms, the tube was sealed and the ligand-containing arm immersed in a bath at 333 K, while the other was left at ambient temperature. After 3 d, crystals had deposited in the arm held at ambient temperature. They were filtered off, washed with acetone and ether, and air dried. Yield: 85%. M.p. = 530 K.

### Refinement

H-atoms were placed in calculated positions [C—H 0.95 Å, *U*_iso_(H) = 1.2*U*_eq_(C)] and were included in the refinement in the riding model approximation.

### Crystal data

**Table:**

[Cu_2_(C_2_Cl_3_O_2_)_2_Cl_2_(C_12_H_8_N_2_)_2_]	*F*(000) = 876
*M**_r_* = 883.15	*D*_x_ = 1.893 Mg m^−^^3^
Monoclinic, *P*2_1_/*n*	Cu *K*α radiation, λ = 1.54184 Å
Hall symbol: -P 2yn	Cell parameters from 7160 reflections
*a* = 9.2961 (2) Å	θ = 4.6–76.4°
*b* = 17.3529 (2) Å	µ = 8.43 mm^−^^1^
*c* = 10.6201 (2) Å	*T* = 100 K
β = 115.269 (3)°	Chip, blue
*V* = 1549.25 (5) Å^3^	0.15 × 0.10 × 0.05 mm
*Z* = 2	

### Data collection

**Table:**

Agilent SuperNova Dual diffractometer with Atlas detector	3233 independent reflections
Radiation source: SuperNova (Cu) X-ray Source	3049 reflections with *I* > 2σ(*I*)
Mirror monochromator	*R*_int_ = 0.019
Detector resolution: 10.4041 pixels mm^-1^	θ_max_ = 76.6°, θ_min_ = 5.1°
ω scans	*h* = −6→11
Absorption correction: multi-scan (*CrysAlis PRO*; Agilent, 2010)	*k* = −21→21
*T*_min_ = 0.365, *T*_max_ = 0.678	*l* = −13→11
11808 measured reflections	

### Refinement

**Table:**

Refinement on *F*^2^	Primary atom site location: structure-invariant direct methods
Least-squares matrix: full	Secondary atom site location: difference Fourier map
*R*[*F*^2^ > 2σ(*F*^2^)] = 0.026	Hydrogen site location: inferred from neighbouring sites
*wR*(*F*^2^) = 0.070	H-atom parameters constrained
*S* = 1.05	*w* = 1/[σ^2^(*F*_o_^2^) + (0.0394*P*)^2^ + 1.203*P*] where *P* = (*F*_o_^2^ + 2*F*_c_^2^)/3
3233 reflections	(Δ/σ)_max_ = 0.001
208 parameters	Δρ_max_ = 0.52 e Å^−^^3^
0 restraints	Δρ_min_ = −0.46 e Å^−^^3^

### Fractional atomic coordinates and isotropic or equivalent isotropic displacement parameters (Å^2^)

**Table:**

	*x*	*y*	*z*	*U*_iso_*/*U*_eq_	
Cu	0.39736 (3)	0.560830 (15)	0.55506 (3)	0.01748 (9)	
Cl1	0.34122 (6)	0.52569 (3)	0.33194 (5)	0.02267 (11)	
Cl2	0.62010 (6)	0.73740 (3)	0.34999 (5)	0.02598 (11)	
Cl3	0.39347 (5)	0.84979 (2)	0.34988 (5)	0.02186 (11)	
Cl4	0.65647 (5)	0.80961 (3)	0.60933 (5)	0.02227 (11)	
O1	0.51331 (16)	0.65057 (7)	0.53709 (14)	0.0210 (3)	
O2	0.28218 (16)	0.71570 (8)	0.44642 (15)	0.0239 (3)	
N1	0.24794 (19)	0.48328 (9)	0.57670 (16)	0.0185 (3)	
N2	0.41913 (18)	0.59423 (9)	0.74473 (16)	0.0178 (3)	
C1	0.2369 (2)	0.49090 (10)	0.70005 (19)	0.0169 (3)	
C2	0.1599 (2)	0.42989 (11)	0.4880 (2)	0.0221 (4)	
H2	0.1681	0.4236	0.4025	0.027*	
C3	0.0548 (2)	0.38227 (11)	0.5162 (2)	0.0248 (4)	
H3	−0.0075	0.3450	0.4496	0.030*	
C4	0.0421 (2)	0.38958 (11)	0.6400 (2)	0.0237 (4)	
H4	−0.0289	0.3578	0.6598	0.028*	
C5	0.1367 (2)	0.44524 (11)	0.7372 (2)	0.0202 (4)	
C6	0.1352 (2)	0.45803 (12)	0.8702 (2)	0.0241 (4)	
H6	0.0657	0.4284	0.8956	0.029*	
C7	0.2308 (2)	0.51143 (12)	0.9603 (2)	0.0242 (4)	
H7	0.2299	0.5173	1.0489	0.029*	
C8	0.3335 (2)	0.55923 (11)	0.9239 (2)	0.0198 (4)	
C9	0.4369 (2)	0.61606 (12)	1.0113 (2)	0.0232 (4)	
H9	0.4454	0.6235	1.1028	0.028*	
C10	0.5252 (2)	0.66045 (11)	0.9628 (2)	0.0241 (4)	
H10	0.5952	0.6987	1.0209	0.029*	
C11	0.5113 (2)	0.64899 (11)	0.8271 (2)	0.0214 (4)	
H11	0.5696	0.6813	0.7934	0.026*	
C12	0.3321 (2)	0.54982 (10)	0.79196 (19)	0.0172 (3)	
C13	0.4236 (2)	0.70725 (10)	0.47750 (19)	0.0182 (4)	
C14	0.5177 (2)	0.77358 (11)	0.44580 (19)	0.0184 (3)	

### Atomic displacement parameters (Å^2^)

**Table:**

	*U*^11^	*U*^22^	*U*^33^	*U*^12^	*U*^13^	*U*^23^
Cu	0.02157 (16)	0.01456 (15)	0.01898 (15)	−0.00160 (10)	0.01120 (12)	−0.00039 (9)
Cl1	0.0286 (2)	0.0222 (2)	0.0181 (2)	0.00446 (17)	0.01078 (18)	0.00221 (16)
Cl2	0.0307 (2)	0.0248 (2)	0.0302 (2)	−0.00004 (18)	0.0204 (2)	−0.00146 (18)
Cl3	0.0233 (2)	0.0184 (2)	0.0213 (2)	0.00170 (16)	0.00706 (18)	0.00467 (16)
Cl4	0.0216 (2)	0.0225 (2)	0.0189 (2)	−0.00312 (16)	0.00503 (17)	0.00003 (16)
O1	0.0221 (7)	0.0157 (6)	0.0266 (7)	0.0007 (5)	0.0118 (6)	0.0035 (5)
O2	0.0188 (7)	0.0208 (7)	0.0320 (7)	−0.0012 (5)	0.0108 (6)	0.0019 (6)
N1	0.0209 (7)	0.0157 (7)	0.0190 (7)	−0.0006 (6)	0.0086 (6)	0.0005 (6)
N2	0.0171 (7)	0.0166 (7)	0.0209 (7)	−0.0009 (6)	0.0091 (6)	−0.0021 (6)
C1	0.0169 (8)	0.0159 (8)	0.0177 (8)	0.0014 (7)	0.0072 (7)	0.0012 (7)
C2	0.0250 (10)	0.0192 (9)	0.0196 (9)	−0.0025 (7)	0.0072 (8)	−0.0030 (7)
C3	0.0226 (9)	0.0186 (9)	0.0276 (10)	−0.0034 (7)	0.0054 (8)	−0.0027 (7)
C4	0.0203 (9)	0.0185 (9)	0.0313 (10)	−0.0018 (7)	0.0100 (8)	0.0025 (8)
C5	0.0187 (9)	0.0175 (9)	0.0236 (9)	0.0008 (7)	0.0082 (8)	0.0040 (7)
C6	0.0254 (10)	0.0244 (9)	0.0273 (10)	0.0016 (8)	0.0157 (9)	0.0063 (8)
C7	0.0280 (10)	0.0263 (10)	0.0226 (9)	0.0039 (8)	0.0149 (8)	0.0032 (8)
C8	0.0192 (9)	0.0203 (9)	0.0190 (9)	0.0046 (7)	0.0074 (7)	0.0001 (7)
C9	0.0210 (9)	0.0254 (10)	0.0212 (9)	0.0051 (7)	0.0072 (8)	−0.0047 (7)
C10	0.0183 (9)	0.0230 (9)	0.0276 (10)	0.0010 (7)	0.0064 (8)	−0.0087 (8)
C11	0.0182 (9)	0.0180 (9)	0.0284 (10)	−0.0005 (7)	0.0103 (8)	−0.0046 (7)
C12	0.0162 (8)	0.0164 (8)	0.0195 (8)	0.0016 (7)	0.0080 (7)	0.0003 (7)
C13	0.0219 (9)	0.0163 (8)	0.0175 (8)	−0.0015 (7)	0.0094 (7)	−0.0017 (7)
C14	0.0185 (8)	0.0180 (8)	0.0191 (8)	0.0007 (7)	0.0083 (7)	0.0007 (7)

### Geometric parameters (Å, º)

**Table:**

Cu—O1	1.9491 (13)	C2—H2	0.9500
Cu—N1	2.0163 (16)	C3—C4	1.375 (3)
Cu—N2	2.0214 (16)	C3—H3	0.9500
Cu—Cl1	2.2811 (5)	C4—C5	1.413 (3)
Cu—Cl1^i^	2.6666 (5)	C4—H4	0.9500
Cl1—Cu^i^	2.6666 (5)	C5—C6	1.436 (3)
Cl2—C14	1.7785 (19)	C6—C7	1.356 (3)
Cl3—C14	1.7652 (19)	C6—H6	0.9500
Cl4—C14	1.7772 (19)	C7—C8	1.437 (3)
O1—C13	1.270 (2)	C7—H7	0.9500
O2—C13	1.220 (2)	C8—C12	1.405 (3)
N1—C2	1.326 (2)	C8—C9	1.413 (3)
N1—C1	1.363 (2)	C9—C10	1.375 (3)
N2—C11	1.328 (2)	C9—H9	0.9500
N2—C12	1.359 (2)	C10—C11	1.405 (3)
C1—C5	1.402 (3)	C10—H10	0.9500
C1—C12	1.430 (2)	C11—H11	0.9500
C2—C3	1.406 (3)	C13—C14	1.567 (3)
			
O1—Cu—N1	168.83 (6)	C4—C5—C6	124.07 (18)
O1—Cu—N2	92.53 (6)	C7—C6—C5	121.42 (18)
N1—Cu—N2	81.87 (6)	C7—C6—H6	119.3
O1—Cu—Cl1	90.15 (4)	C5—C6—H6	119.3
N1—Cu—Cl1	94.40 (5)	C6—C7—C8	120.98 (18)
N2—Cu—Cl1	173.20 (5)	C6—C7—H7	119.5
O1—Cu—Cl1^i^	93.33 (4)	C8—C7—H7	119.5
N1—Cu—Cl1^i^	96.47 (5)	C12—C8—C9	116.77 (18)
N2—Cu—Cl1^i^	91.64 (5)	C12—C8—C7	118.51 (18)
Cl1—Cu—Cl1^i^	94.440 (17)	C9—C8—C7	124.72 (18)
Cu—Cl1—Cu^i^	85.560 (17)	C10—C9—C8	119.49 (18)
C13—O1—Cu	113.24 (12)	C10—C9—H9	120.3
C2—N1—C1	118.26 (16)	C8—C9—H9	120.3
C2—N1—Cu	129.20 (13)	C9—C10—C11	119.75 (18)
C1—N1—Cu	112.51 (12)	C9—C10—H10	120.1
C11—N2—C12	118.75 (16)	C11—C10—H10	120.1
C11—N2—Cu	128.60 (13)	N2—C11—C10	121.87 (18)
C12—N2—Cu	112.52 (12)	N2—C11—H11	119.1
N1—C1—C5	123.17 (17)	C10—C11—H11	119.1
N1—C1—C12	116.54 (16)	N2—C12—C8	123.27 (17)
C5—C1—C12	120.28 (17)	N2—C12—C1	116.50 (16)
N1—C2—C3	122.30 (18)	C8—C12—C1	120.23 (17)
N1—C2—H2	118.9	O2—C13—O1	129.09 (18)
C3—C2—H2	118.9	O2—C13—C14	119.30 (16)
C4—C3—C2	120.00 (18)	O1—C13—C14	111.59 (16)
C4—C3—H3	120.0	C13—C14—Cl3	112.75 (13)
C2—C3—H3	120.0	C13—C14—Cl2	110.35 (12)
C3—C4—C5	118.77 (18)	Cl3—C14—Cl2	108.20 (10)
C3—C4—H4	120.6	C13—C14—Cl4	106.71 (12)
C5—C4—H4	120.6	Cl3—C14—Cl4	108.88 (10)
C1—C5—C4	117.49 (18)	Cl2—C14—Cl4	109.93 (10)
C1—C5—C6	118.44 (18)		
			
O1—Cu—Cl1—Cu^i^	93.35 (4)	C3—C4—C5—C1	1.0 (3)
N1—Cu—Cl1—Cu^i^	−96.86 (5)	C3—C4—C5—C6	−179.53 (19)
Cl1^i^—Cu—Cl1—Cu^i^	0.0	C1—C5—C6—C7	−1.7 (3)
N1—Cu—O1—C13	−31.8 (4)	C4—C5—C6—C7	178.81 (19)
N2—Cu—O1—C13	−91.39 (13)	C5—C6—C7—C8	2.2 (3)
Cl1—Cu—O1—C13	82.36 (12)	C6—C7—C8—C12	0.4 (3)
Cl1^i^—Cu—O1—C13	176.82 (12)	C6—C7—C8—C9	−179.89 (19)
O1—Cu—N1—C2	117.1 (3)	C12—C8—C9—C10	2.3 (3)
N2—Cu—N1—C2	177.57 (18)	C7—C8—C9—C10	−177.43 (19)
Cl1—Cu—N1—C2	3.29 (17)	C8—C9—C10—C11	0.2 (3)
Cl1^i^—Cu—N1—C2	−91.69 (17)	C12—N2—C11—C10	2.1 (3)
O1—Cu—N1—C1	−60.8 (4)	Cu—N2—C11—C10	−173.53 (14)
N2—Cu—N1—C1	−0.31 (13)	C9—C10—C11—N2	−2.5 (3)
Cl1—Cu—N1—C1	−174.59 (12)	C11—N2—C12—C8	0.5 (3)
Cl1^i^—Cu—N1—C1	90.43 (12)	Cu—N2—C12—C8	176.88 (14)
O1—Cu—N2—C11	−12.19 (17)	C11—N2—C12—C1	−179.01 (16)
N1—Cu—N2—C11	177.52 (17)	Cu—N2—C12—C1	−2.7 (2)
Cl1^i^—Cu—N2—C11	81.21 (16)	C9—C8—C12—N2	−2.7 (3)
O1—Cu—N2—C12	171.92 (13)	C7—C8—C12—N2	177.00 (17)
N1—Cu—N2—C12	1.63 (12)	C9—C8—C12—C1	176.80 (17)
Cl1^i^—Cu—N2—C12	−94.68 (12)	C7—C8—C12—C1	−3.5 (3)
C2—N1—C1—C5	−0.2 (3)	N1—C1—C12—N2	2.5 (2)
Cu—N1—C1—C5	177.90 (14)	C5—C1—C12—N2	−176.45 (16)
C2—N1—C1—C12	−179.17 (17)	N1—C1—C12—C8	−177.04 (16)
Cu—N1—C1—C12	−1.0 (2)	C5—C1—C12—C8	4.0 (3)
C1—N1—C2—C3	1.0 (3)	Cu—O1—C13—O2	9.5 (3)
Cu—N1—C2—C3	−176.75 (14)	Cu—O1—C13—C14	−171.79 (11)
N1—C2—C3—C4	−0.8 (3)	O2—C13—C14—Cl3	−6.2 (2)
C2—C3—C4—C5	−0.3 (3)	O1—C13—C14—Cl3	174.97 (13)
N1—C1—C5—C4	−0.8 (3)	O2—C13—C14—Cl2	−127.31 (16)
C12—C1—C5—C4	178.13 (17)	O1—C13—C14—Cl2	53.85 (18)
N1—C1—C5—C6	179.71 (17)	O2—C13—C14—Cl4	113.29 (17)
C12—C1—C5—C6	−1.4 (3)	O1—C13—C14—Cl4	−65.55 (17)

Symmetry code: (i) −*x*+1, −*y*+1, −*z*+1.

### Hydrogen-bond geometry (Å, º)

**Table:**

*D*—H···*A*	*D*—H	H···*A*	*D*···*A*	*D*—H···*A*
C2—H2···Cl3^ii^	0.95	2.80	3.679 (2)	154
C4—H4···O2^iii^	0.95	2.49	3.302 (3)	144
C7—H7···Cl1^iv^	0.95	2.73	3.638 (2)	159

Symmetry codes: (ii) −*x*+1/2, *y*−1/2, −*z*+1/2; (iii) −*x*, −*y*+1, −*z*+1; (iv) *x*, *y*, *z*+1.
